# Cardiac Rupture—The Most Serious Complication of Takotsubo Syndrome: A Series of Five Cases and a Systematic Review

**DOI:** 10.3390/jcm10051066

**Published:** 2021-03-04

**Authors:** Małgorzata Zalewska-Adamiec, Hanna Bachórzewska-Gajewska, Sławomir Dobrzycki

**Affiliations:** 1 Department of Invasive Cardiology, Medical University of Bialystok, 15-276 Bialystok, Poland; hgajewska@op.pl (H.B.-G.); kki@umb.edu.pl (S.D.); 2 Department of Clinical Medicine, Medical University of Bialystok, 15-295 Bialystok, Poland

**Keywords:** Takotsubo syndrome, cardiac rupture, cardiac perforation, acute coronary syndrome, cardiac tamponade

## Abstract

Background: The most serious complication of the acute Takotsubo phase is a myocardial perforation, which is rare, but it usually results in the death of the patient. Methods: In the years 2008–2020, 265 patients were added to the Podlasie Takotsubo Registry. Cardiac rupture was observed in five patients (1.89%), referred to as the Takotsubo syndrome with complications of cardiac rupture (TS+CR) group. The control group consisted of 50 consecutive patients with uncomplicated TS. The diagnosis of TS was based on the Mayo Clinic Criteria. Results: Cardiac rupture was observed in women with TS aged 74–88 years. Patients with TS and CR were older (82.20 vs. 64.84; *p* = 0.011), than the control group, and had higher troponin, creatine kinase, aspartate aminotransferase, and blood glucose levels (168.40 vs. 120.67; *p* = 0.010). The TS+CR group demonstrated a higher heart rate (95.75 vs. 68.38; *p* < 0.0001) and the Global Registry of Acute Coronary Events (GRACE) scores (186.20 vs. 121.24; *p* < 0.0001) than the control group. In patients with CR, ST segment elevation was recorded significantly more often in the III, V4, V5 and V6 leads. Left ventricular free wall rupture was noted in four patients, and in one case, rupture of the ventricular septum. In a multivariate logistic regression, the factors that increase the risk of CR in TS were high GRACE scores, and the presence of ST segment elevation in lead III. Conclusions: Cardiac rupture in TS is rare but is the most severe mechanical complication and is associated with a very high risk of death. The main risk factors for left ventricular perforation are female gender, older age, a higher concentration of cardiac enzymes, higher GRACE scores, and ST elevations shown using electrocardiogram (ECG).

## 1. Introduction

Takotsubo syndrome (TS), first described in Japan around 30 years ago by Sato et al. [1], is a stress-induced transient impairment of left ventricular contractility with a concomitant increase in the level of cardiac enzymes and ischemic changes in electrocardiograms and with no significant atherosclerotic lesions in the coronary arteries. The clinical manifestation of TS is similar to that of an acute coronary syndrome (ACS). Its prognosis is usually benign, and the contractility disorders disappear within a few weeks to several months after the event. However, in the acute state, approximately 20% of patients have serious complications. The most frequently occurring complication is acute heart failure manifested by cardiogenic shock or pulmonary oedema. Cardiac rhythm and conduction disorders, including sudden cardiac arrest, are also frequently recorded. However, the most serious complication of the acute Takotsubo phase is a myocardial perforation, which is rare, but it usually results in the death of the patient [2,3,4,5].

The aim of this study was to identify the risk factors of cardiac rupture (CR) in Takotsubo syndrome. A systematic review of previously reported cases of CR in Takotsubo syndrome was also performed.

## 2. Materials and Methods

### 2.1. Study Population

In the years 2008–2020, 265 patients were added to the Podlasie Takotsubo Registry. Cardiac rupture was observed in five patients (1.89%), referred to as the Takotsubo syndrome with complications of cardiac rupture (TS+CR) group. The control group consisted of 50 consecutive patients with uncomplicated Takotsubo syndrome, hospitalized at the authors’ clinic.

In the analyzed patients, Takotsubo syndrome was diagnosed on the basis of the Mayo Clinic criteria [6], in force during their hospitalization. Cardiac rupture was diagnosed in imaging tests (i.e., echocardiography and ventriculography).

### 2.2. Systematic Review

For a systematic review, all Takotsubo syndrome cardiac rupture cases were searched in PubMed; 35 cases were found published between 2004 and 2020. The compilation took into account the age, sex, place of left ventricular perforation, and the patient’s survival/death. The treatment was taken into account in the surviving patients.

### 2.3. Statistical Analysis

Both the test and control group data were statistically analyzed. We compared the quantitative variables using the Student’s *t*-test and the Mann–Whitney *U*-test and compared the qualitative data using the chi-square and Fisher tests. Multivariate analysis was performed using logistic regression. A *p*-value of less than 0.05 was considered statistically significant. The analysis was performed using STATISTICA software, version 13.3 (StatSoft Poland, Cracow, Poland).

## 3. Results

### 3.1. General Characteristics

Cardiac rupture in Takotsubo syndrome was observed in women in the 74–88-year age group (average = 82.2 years). The Takotsubo syndrome patients with complications of CR (the TS+CR group) were older than the patients in the control group. In all five women in the TS+CR group, the main symptom of Takotsubo syndrome was retrosternal pain. Laboratory tests performed on admission in the TS+CR group showed higher values of troponin and creatine kinase than that of the control group; however, the differences were not statistically significant. The values obtained for glycemia were significantly higher for the TS+CR group than that of the control group (Table 1). The values obtained for glycated hemoglobin (HbA_1C_) in the TS+CR group were normal (5.1–5.6%).

Patients in the TS+CR group demonstrated a significantly higher heart rate than that of the control group. In addition, the scores on the Global Registry of Acute Coronary Events (GRACE) scale were significantly higher for the TS+CR group than for the control group (Table 1).

In all five patients of the TS+CR group, the Takotsubo apical variant was diagnosed, including two patients with left ventricular middle segment involvement. In the control group, the apical variant was found in 49 patients (98%), including 15 with middle segment involvement. The focal-type TS was found in one patient.

### 3.2. ECG—ST Segment Elevation

**Figure 1:**
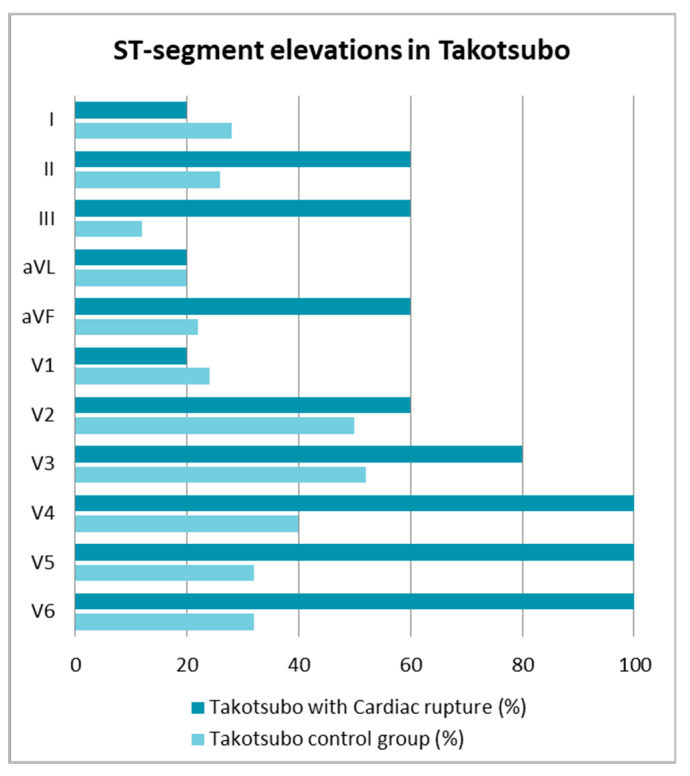
Comparison of ST segment elevations in the TS groups.

ST segment elevations were observed in all five patients in the TS+CR group on admission, and 66% in the control group. In all patients with CR, ST elevations were located in the V4, V5 and V6 leads, while in three patients, in leads from the lower wall as well. Compared to the control group, in patients with CR, ST elevation was significantly more often recorded in leads III, V4, V5, and V6 (Figure 1 and Table 2).

### 3.3. Cardiac Rupture

In one patient, CR occurred on the first day of hospitalization, which was detected during coronarography with ventriculography. In two patients, CR occurred on the second day of hospitalization, and in the other two patients, CR occurred on the fifth day of hospitalization. In these four patients, CR was diagnosed during echocardiography—in two after cardiac arrest during resuscitation, and in two due to symptoms of cardiogenic shock. The left ventricular free wall rupture was noted in four patients, and in one case, it was the rupture of the ventricular septum with a left–right shunt. The patient whose CR was detected during coronarography was cardio-surgically treated and survived, and the remaining patients died (Table 3).

In multivariate logistic regression, the factors that increase the risk of CR in TS are high GRACE scores, and the presence of ST segment elevation in lead III. Table 4 shows the results of our analyses.

### 3.4. Systematic Review

Table 5 shows all case descriptions on CR in TS found in the PubMed database.

## 4. Discussion

Left ventricular wall rupture is undoubtedly the most serious mechanical complication of both myocardial infarction and Takotsubo syndrome. Due to the increasing tamponade, without rapid cardiac intervention, most patients die. The introduction of an effective treatment for the causal myocardial infarction with percutaneous coronary angioplasty has significantly reduced the incidence of cardiac rupture, which is now detected in only 2–4% of the patients with myocardial infarction [42]. Usually, cardiac rupture occurs in older patients with myocardial infarction who have been treated with conservative or delayed coronary angioplasty, and the rupture site is within the necrotic wall.

Left ventricular wall perforation in the course of Takotsubo syndrome is also usually fatal, but it occurs rarely, in approximately 1% of patients [5,43,44]. In our register, the percentage of CR during TS is slightly higher (1.89%). The pathophysiological mechanism of cardiac rupture in Takotsubo syndrome is not yet fully understood. Autopsy studies have thus far described the atrophy of myocyte striations and features of inflammation with foci of necrosis in the area of the ventricular wall rupture [8,14,22,39].

In 2011, Kumar et al. [5] conducted the first systematic review of all cases of CR in TS available in the MEDLINE database from 1950 to 2009, and collected 12 patients reported from 2004 to 2009. The group, as in our analysis, was made up of women themselves, aged 62–90 (average age = 76). Rupture of the ventricular septum was detected in two patients, perforation in the right ventricular septum in two women, and perforation in the left ventricular free wall in seven women. In the case of one patient, the site of perforation was not reported. Out of the 12 patients included in the review of Kumar et al., 10 patients (83%) died, including half of them in the first two days of hospitalization; the remaining patients died in the next eight days. Such high mortality rates obtained in the systematic review and in this study (80%) are comparable to the deaths of patients with cardiac rupture during myocardial infarction not treated with cardiac surgery [45].

Another review of Takotsubo syndrome complicated by cardiac rupture was performed by Dalia et al. [38]. They analyzed 20 cases from the MEDLINE database with a rupture of the left ventricular free wall. The majority of these patients were women (95%) with an average age of 74.9 years. Dalia et al. reported a mortality rate of 85%.

Currently, the PubMed database contains 35 case studies on CR in Takotsubo syndrome. Most patients were women, but there were also three men. The youngest patient was 57 years old, and the oldest was 92 years; 29 patients were aged over 70 years. The free wall of the left ventricle ruptured in 22 patients, the free wall of the right ventricle ruptured in three patients, and perforation in the ventricular septum was recorded in 10 cases. Out of 35 patients, 23 died; 17 patients with a rupture of the free wall of the left ventricle (77.3%), three women with a rupture of the right ventricle (100%), and three patients with a rupture of the interventricular septum (30%). A total of 12 patients survived, including five patients with left ventricular free wall rupture, of whom four patients were treated with cardiac surgery and one patient was treated with conservative treatment. In addition, seven patients with ventricular septal perforation survived, six of whom were treated surgically and one was treated conservatively.

In this study, out of the five patients in the TS+CR group, four people (80%) died. Among them, CR occurred on the second and fifth days of hospitalization. One patient survived thanks to cardiac surgery. The data in the literature on the survival of patients with CR in TS show that the prognosis of patients with ventricular septal perforation is better. In published cases, the ruptured septum was supplied by cardiac surgery or closed by an occlude [30,37,39]. Izumi et al. [15] described the history of a 73-year-old patient with stress cardiomyopathy, who had a perforated ventricular septum on the first day of hospitalization. The patient survived, although she was operated on only on day 22 after the rupture of the ventricular septum. The prognosis is much worse in patients with the rupture of the free wall of the left or, less frequently, of the right ventricle. Out of 25 patients from the PubMed database, 20 people died, whereas the patients undergoing cardiac surgery survived.

In the TS+CR group, there were significantly higher values for glycemia. These patients did not suffer from diabetes and the values of HbA_1C_ were normal. Therefore, hyperglycemia should be explained by the high secretion of catecholamines.

The etiology of cardiac rupture in Takotsubo syndrome is undoubtedly multifactorial. According to Kumar et al., the factors conducive to left ventricular perforation are female gender, older age, higher arterial pressure, presence of ST elevation in the inferior wall leads, low left ventricular ejection fraction, and high peak left ventricular systolic fraction. Our analysis of patients with CR in TS compared to the control group undoubtedly shows that the factors that increase the risk of CR in TS are older age, a higher concentration of cardiac enzymes, higher GRACE scores, faster heart rate, and ST elevation in ECG, especially in leads V4–V6 and the inferior wall leads.

The analyzed series of five cases, as well as those quoted in the data in the literature, prove the importance of the monitoring and observation of patients with TS and risk factors for CR during the first few days of diagnosis of TS. Only a quick diagnosis of perforation, decompression of the subsequent tamponade, and urgent cardiac surgery offer these patients a chance to survive.

### Limitations

The primary limitation of this study is the small number of patients—only five patients with a CR were studied, due to the rarity of this complication and the limitation of the Takotsubo syndrome register to the Podlaskie Voivodeship.

## 5. Conclusions and Clinical Implications

Cardiac rupture in Takotsubo syndrome is rare but is the most severe mechanical complication and is associated with a very high risk of death. Female gender, older age, a higher concentration of cardiac enzymes, a higher number of points in GRACE scale, a faster heart rate, and ST elevations in ECG, especially in the lower and anterior wall ventricular leads, are the main risk factors for left ventricular perforation. Only intensive monitoring in the acute phase of the disease allows for a quick diagnosis of CR, and urgent surgical intervention offers these patients a chance to survive.

## Figures and Tables

**Table 1:** Results comparison of the two groups.

	TS+CR Group *n* = 5	TS Control Group *n* = 50	*p*-Value
Clinical and demographic characteristics:			
Age (years)	82.20 ± 5.67	64.84 ± 14.51	0.011
Female sex (%)	5 (100)	46 (92)	0.511
Body mass index (BMI) (kg/m^2^)	24.02 ± 2.83	26.23 ± 5.27	0.415
Trigger factor:			
Physical (%)	0 (0)	12 (24)	0.215
Emotional (%)	3 (60)	17 (34)	0.249
Absent/unknown (%)	2 (40)	21 (42)	0.931
History of hypertension (%)	4 (80)	30 (60)	0.380
Hyperlipidemia (%)	2 (40)	23 (46)	0.797
Smoking (%)	0 (0)	14 (28)	0.171
Family history of coronary artery disease (%)	0 (0)	13 (26)	0.192
Diabetes mellitus (%)	0 (0)	7 (14)	0.371
Anxiety/depression (%)	1 (20)	5 (10)	0.494
Thyroid disorders (%)	0 (0)	14 (28)	0.171
Chronic kidney disease (%)	3 (60)	12 (24)	0.084
COPD (%)	0 (0)	6 (12)	0.412
CHA2DS2-VASc scale (points)	4.00 ((0.00))	3.00 ((2.00))	0.138
GRACE scale (points)	186.20 ± 22.62	121.24 ± 34.57	0.0001
Diagnostic tests (echocardiography, coronarography, and ECG):			
Left ventricular ejection fraction on admission (%)	38.60 ± 7.89	39.94 ± 10.52	0.783
Normal coronary arteries (%)	1 (20)	25 (50)	0.202
Non-significant stenoses (%)	4 (80)	25 (50)	0.202
ECG—ST segment elevation (%)	5 (100)	33 (66)	0.116
QTc on admission (ms)	468.50 ± 52.22	475.44 ± 38.98	0.747
QTc after a few days (ms)	525.00 ((157.00))	508.00 ((37.00))	0.429
Laboratory parameters:			
Hemoglobin (mg/dL)	12.62 ± 0.92	13.45 ± 1.73	0.295
Erythrocytes (×106/μL)	4.28 ((0.45))	4.44 ((0.59))	0.151
Hematocrit (%)	36.30 ((2.30))	39.40 ((5.60))	0.229
Leukocytes (×103/μL)	11.66 ± 2.80	9.52 ± 3.69	0.213
Glucose on admission (mg/dL)	160.00 ((50.00))	112.00 ((36.00))	0.015
Creatinine (mg/dL)	0.90 ((0.18))	0.75 ((0.26))	0.395
eGFR MDRD (mL/min/1.72 m^2^)	62.37 ± 14.85	74.69 ± 22.24	0.233
CK (IU/L)	275.00 ((574.50))	199.50 ((203.00))	0.126
Troponin (significant increase) (%)	5 (100)	48 (96)	0.648
Troponin—mean concentration (ng/mL)	2.59 ((7.03))	2.13 ((3.70))	0.726
AspAT (mg/dL)	53.50 ((82.50))	32.00 ((27.00))	0.276
AlAT (mg/dL)	23.00 ((69.00))	21.00 ((13.00))	0.775
Total cholesterol (mg/dL)	171.50 ± 25.49	186.06 ± 42.61	0.506
LDL (mg/dL)	97.45 ± 37.67	117.62 ± 38.82	0.323
HDL (mg/dL)	61.55 ± 11.09	50.06 ± 18.08	0.219
Triglycerides (mg/dL)	53.00 ((18.75))	79.00 ((58.00))	0.056
CRP (mg/L)	13.70 ((40.20))	20.35 ((27.30))	0.933
BNP (pg/mL)	974.65 (711.7)	464.00 (260.71)	0.058
Clinical course and mortality:			
BPs on admission	123.00 ± 36.51	123.28 ± 23.94	0.981
HR on admission	95.75 ± 22.46	68.38 ± 11.42	<0.0001
Retrosternal chest pain (%)	5 (100)	41 (82)	0.299
Dyspnea (%)	0 (0)	6 (12)	0.411
Killip class III/IV on admission (%)	1 (20)	5 (10)	0.494
Pneumonia (%)	1 (20)	13 (26)	0.769
Rhythm disturbances (%)	0 (0)	4 (8)	0.511
Hospital mortality (%)	4 (80)	4 (8)	<0.0001
Pharmacological treatment:			
Unfractionated heparin (%)	1 (20)	17 (34)	0.524
Enoxaparin (%)	4 (80)	28 (56)	0.299
Clopidogrel (%)	3 (60)	43 (86)	0.134
Aspirin (%)	5 (100)	49 (98)	0.749
Statin (%)	5 (100)	44 (88)	0.412
Beta blocker (%)	5 (100)	45 (90)	0.458
ACE inhibitor/AT-R blocker (%)	4 (80)	46 (92)	0.374
Diuretics (%)	4 (80)	25 (50)	0.200
Proton pump inhibitor (%)	5 (100)	45 (90)	0.458
Pressor amines (%)	3 (60)	8 (16)	0.019

Data are presented as arithmetic means ± standard deviations, medians ((interquartile interval)), and numbers (percentages). Abbreviations: ACE, angiotensin converting enzyme; AlAT, alanine aminotransferase; AspAT, aspartate aminotransferase; AT-R, angiotensin receptor; BNP, B-type natriuretic peptide; BPs, systolic blood pressure; COPD, chronic obstructive pulmonary disease; CHA2DS2-VASc, congestive heart failure, hypertension, age (>65 = 1 point; >75 = 2 points), diabetes, previous stroke/transient ischemic attack (2 points)-vascular disease; CK, creatine kinase; CRP, C-reactive protein; CR, cardiac rupture; LDL, low-density lipoprotein; GRACE, the Global Registry of Acute Coronary Events; HDL, high-density lipoprotein; HR, heart rate; eGFR MDRD, estimated glomerular filtration rate using modified diet renal disease; ECG, electrocardiogram; SD, standard deviation; TS, Takotsubo syndrome; TS+CR, Takotsubo syndrome with complications of cardiac rupture.

**Table 2:** ST segment elevations in patients with Takotsubo syndrome and cardiac rupture.

Case	I	II	III	aVL	aVF	V1	V2	V3	V4	V5	V6
Case 1							+	+	+	+	+
Case 2		+	+		+		+	+	+	+	+
Case 3	+			+		+	+	+	+	+	+
Case 4		+	+		+				+	+	+
Case 5		+	+		+			+	+	+	+
TS+CR (%)	20	60	60	20	60	20	60	80	100	100	100
TS control group (%)	28	26	12	20	22	24	50	52	40	32	32
*p*-Value	0.701	0.110	0.005	—	0.062	0.841	0.669	0.231	0.010	0.002	0.002

Data are presented as percentages. Abbreviation: TS+CR, Takotsubo syndrome with complications of cardiac rupture.

**Table 3:** Clinical characteristics and imaging of the five cases.

Case	Age (Years)	Sex (F/M)	Symptoms at Admission	ECG at Admission	ECHO at Admission	LVEF ECHO	Coronarography	Ventriculography	Last ECHO	CR—Day of Hospitalization
Case 1	74	F	Retrosternal pain, cardiogenic shock	SR 58/min, elevation ST in leads V2–V6	Akinesis of the apex with hyperkinesis of the other LV wall segments, tamponade	55%	Mural atherosclerotic lesions in the coronary arteries	EF 56%Visible contrast leakage around the LV apex	EF—50% (discharge)	Day 1
Case 2	79	F	Retrosternal pain	SR 50/min, ST elevation with negative T waves in II, III, aVF, and V2–V6	Akinesis of the apex and apical LV wall segments	43%	No atherosclerotic lesions in the coronary arteries	Akinesis aneurysm of the LV apex, EF—40%	LV free wall rupture, tamponade	Day 2—Death
Case 3	84	F	Restosternal pain, cardiogenic shock	SR 90/min, ST elevation in I, aVL, and V1–V6	Akinesis of the apex and apical and central LV wall segments	30%	Mural atherosclerotic lesions	Not performed	VSD with left–right shut	Day 2 Death on Day 5
Case 4	88	F	Restosternal pain	FA 71/min, ST elevation in II, III, aVF, V4–V6, and negative T in V4–V6	A/dyskinesis of the apex and apical LV wall segments	38%	40%–50% RCA, mural lesions in others	EF—32%	LV free wall rupture, tamponade	Day 5—Death
Case 5	85	F	Restosternal pain	FA 108/min, ST elevations in II, III, aVF, V3–V6, negative T in I, II, III, aVL, aVF, and V3–V6	A/dyskinesis of the apex and apical LV wall segments, hypokinesis of central segment	35%	80% 1D, insignificant lesions in others	EF—35%	LV free wall rupture, tamponade	Day 5—Death

Abbreviations: CR, cardiac rupture; D1, diagonal branch; ECG, electrocardiogram; ECHO, echocardiogram; EF, ejection fraction; FA, atrial fibrillation; F/M, female/male; LV, left ventricle; LVEF, left ventricular ejection fraction; RCA, right coronary artery; SR, sinus rhythm; VSD, ventricular septal defect.

**Table 4:** Results of the univariate and multivariate logistic regression analyses—risk factors for cardiac rupture in Takotsubo syndrome.

	Univariate			Multivariate		
Predictor	Odds Ratio	95% CI	*p*-Value	Odds Ratio	95% CI	*p*-Value
Age (years)	1.229	1.035–1.459	0.0187			
Emotional trigger	2.912	0.443–19.130	0.266			
BPs	1.000	0.963–1.038	0.9807			
Hypertension	2.667	0.277–25.637	0.3956			
HR	0.964	0.911–1.020	0.202			
EF < 40%	0.987	0.901–1.082	0.778			
Hemoglobin	0.670	0.326–1.377	0.276			
ST elevation in III	29.333	2.793–308.040	0.004	25.500	2.278–4334.264	0.017
ST elevation in V5	8.500	0.878–82.315	0.064			
AspAT	1.022	0.998–1.047	0.077			
Glucose	1.017	1.000–1.035	0.051			
LDL	0.984	0.984–1.016	0.323			
GRACE points	1.064	1.014–1.116	0.011	1.064	1.003–1.104	0.036

Abbreviations: CI, confidence interval; BP, blood pressure; HR, heart rate; EF, ejection fraction; LDL, low-density lipoprotein; GRACE, the Global Registry of Acute Coronary Events.

**Table 5:** Reported cases of cardiac rupture in Takotsubo syndrome (*n* = 35).

Author(s) and Year of Publication	Age (Years)	Sex (F/M)	Place of Perforation	Death (+)	Treatment
Akashi et al. in 2004 [7]	70	F	Free wall LV	+	
Sakai et al. in 2005 [8]	84	F	VSD	+	
Ohara et al. in 2005 [9]	79	F	Free wall LV	+	
Ishida et al. in 2005 [10]	67	F	Free wall LV	–	Cardiac surgery
Mafrici et al. in 2006 [11]	87	F	Free wall LV	+	
Yamada et al. in 2006 [12]	71	F	Free wall LV	+	
Shinozaki et al. in 2007 [13]	90	F	Free wall LV	+	
Sacha et al. in 2007 [14]	81	F	Free wall LV	+	
Izumi et al. in 2008 [15]	73	F	VSD	–	Cardiac surgery
Ieva et al. in 2009 [16]	65	F	RV	+	
Stöllberger et al. in 2009 [17]	71	F	Free wall LV	+	
Tsunoda et al. in 2010 [18]	74	F	Free wall LV	+	
Mariscalco et al. in 2010 [19]	71	F	Free wall LV	–	Cardiac surgery
Kurisu et al. in 2012 [20]	81	F	Free wall LV	+	
Jaguszewski et al. in 2012 [21]	82	F	Free wall LV	+	
Yoshida et al. in 2013 [22]	78	F	Free wall LV	–	Conservative
Kumar et al. in 2012 [23]	62	F	Free wall LV	+	
Y-Hassan et al. in 2014 [24]	73	M	Free wall LV	+	
Indorato et al. in 2015 [25]	70	F	Free wall LV	+	
Aikawa et al. in 2015 [26]	81	F	VSD	+	
Showkathali et al. in 2015 [27]	86	F	Free wall LV	+	
Miyake et al. in 2015 [28]	73	M	VSD	+	
Zalewska-Adamiec et al. in 2016 [29]	74	F	Free wall LV	–	Cardiac surgery
Pepe et al. in 2016 [30]	84	F	VSD	–	Occluder
Sung et al. in 2017 [31]	73	F	RV + VSD	+	
Mitchell et al. in 2017 [32]	82	F	RV	+	
Kudaiberdiew et al. in 2017 [33]	63	F	Free wall LV	–	Cardiac surgery
Iskander et al. in 2018 [34]	77	F	Free wall LV	+	
Narita et al. in 2018 [35]	92	M	VSD	–	Conservative
Tsuji et al. in 2018 [36]	71	F	VSD	–	Cardiac surgery
De Manna et al. in 2019 [37]	57	F	VSD	–	Occluder
Dalia et al. in 2019 [38]	75	F	Free wall LV	+	
Zhukova et al. in 2019 [39]	81	F	VSD	–	Occluder
Webster et al. in 2019 [40]	68	F	Free wall LV + VSD	+	
Al-Tkrit et al. in 2020 [41]	77	F	Free wall LV	–	Cardiac surgery

Abbreviations: VSD, ventricular septal defect; RV, right ventricle; LV, left ventricle.

## Data Availability

The data presented in this study are available on request from the corresponding author.

## References

[1] Sato H., Tateishi H., Uchida T., Kodama K., Haze K., Hon M. (1990). Takotsubo-type cardiomyopathy due to multivessel spasm. Clinical Aspect of Myocardial Injury: From Ischemia to Heart Failure.

[2] Ghadri J.R., Wittstein I.S., Prasad A., Sharkey S., Dote K., Akashi Y.J., Cammann V.L., Crea F., Galiuto L., Desmet W. (2018). International Expert Consensus Document on Takotsubo Syndrome (Part I): Clinical Characteristics, Diagnostic Criteria, and Pathophysiology. Eur. Heart J..

[3] Ghadri J.R., Wittstein I.S., Prasad A., Sharkey S., Dote K., Akashi Y.J., Cammann V.L., Crea F., Galiuto L., Desmet W. (2018). International Expert Consensus Document on Takotsubo Syndrome (Part II): Diagnostic Workup, Outcome, and Management. Eur. Heart J..

[4] Nef H.M., Möllmann H., Elsässer A. (2007). Tako-tsubo cardiomyopathy (apical ballooning). Heart.

[5] Kumar S., Kaushik S., Nautiyal A., Choudhary S.K., Kayastha B.L., Mostow N., Lazar J.M. (2011). Cardiac rupture in takotsubo cardiomyopathy: A systematic review. Clin. Cardiol..

[6] Bybee K.A., Kara T., Prasad A., Lerman A., Barsness G.W., Wright R.S., Rihal C.S. (2004). Systematic review: Transient left ventricular apical ballooning: A syndrome that mimics ST-segment elevation myocardial infarction. Ann. Intern. Med..

[7] Akashi Y.J., Tejima T., Sakurada H., Matsuda H., Suzuki K., Kawasaki K., Tsuchiya K., Hashimoto N., Musha H., Sakakibara M. (2004). Left ventricular rupture associated with Takotsubo cardiomyopathy. Mayo Clin. Proc..

[8] Sakai K., Ochiai H., Katayama N., Nakamura K., Arataki K., Kido T., Iwamoto H., Nakamura S., Nakanishi T. (2005). Ventricular septal perforation in a patient with takotsubo cardiomyopathy. Circ. J..

[9] Ohara Y., Hiasa Y., Hosokawa S., Tomokane T., Yamaguchi K., Ogura R., Miyajima H., Ogata T., Yuba K., Suzuki N. (2005). Left ventricular free wall rupture in transient left ventricular apical ballooning. Circ. J..

[10] Ishida T., Yasu T., Arao K., Kawakami M., Saito M. (2005). Bedside diagnosis of cardiac rupture by contrast echocardiography. Circulation.

[11] Mafrici A., Proietti R., Fusco R., De Biase A., Klugmann S. (2006). Left ventricular free wall rupture in a Caucasian female with takotsubo syndrome: A case report and a brief literature review. J. Cardiovasc. Med..

[12] Yamada R., Watanabe N., Kume T., Kawamoto T., Okahashi N., Wada N., Koyama Y., Toyota E., Okura H., Yoshida K. (2006). Left ventricular rupture associated with takotsubo-like left ventricular dysfunction (apical ballooning). J. Echocardiogr..

[13] Shinozaki K., Tamura A., Abe Y., Yano S., Kadota J. (2007). Left ventricular free wall rupture in takotsubo cardiomyopathy. Int. J. Cardiol..

[14] Sacha J., Maselko J., Wester A., Szudrowicz Z., Pluta W. (2007). Left ventricular apical rupture caused by takotsubo cardiomyopathy—Comprehensive pathological heart investigation. Circ. J..

[15] Izumi K., Tada S., Yamada T. (2008). A case of Takotsubo cardiomyopathy complicated by ventricular septal perforation. Circ. J..

[16] Ieva R., Correale M., Brunetti N.D., Di Biase M. (2009). A “bad” case of Tako-Tsubo syndrome. J. Thromb. Thrombolysis.

[17] Stöllberger C., Huber J.O., Enzelsberger B., Finsterer J. (2009). Fatal outcome of epileptic seizure-induced takotsubo syndrome with left ventricular rupture. Eur. J. Neurol..

[18] Tsunoda S., Tando S., Doi T., Kitamura Y., Ogawa M., Tanabe S.I., Yamada C., Yasukawa S., Oda Y. (2010). Left ventricular free wall rupture associated with a combination of acute myocardial infarction and stress-provoked cardiomyopathy: An autopsy case. J. Cardiol. Cases.

[19] Mariscalco G., Cattaneo P., Rossi A., Baravelli M., Piffaretti G., Scannapieco A., Nassiacos D., Sala A. (2010). Tako-tsubo cardiomyopathy complicated by ventricular septal perforation and septal dissection. Heart Vessels.

[20] Kurisu S., Inoue I. (2012). Cardiac rupture in tako-tsubo cardiomyopathy with persistent ST-segment elevation. Int. J. Cardiol..

[21] Jaguszewski M., Fijalkowski M., Nowak R., Czapiewski P., Ghadri J.R., Templin C., Rynkiewicz A. (2012). Ventricular rupture in Takotsubo cardiomyopathy. Eur. Heart. J..

[22] Yoshida S., Miwa K., Matsubara T., Yasuda T., Inoue M., Teramoto R., Okada H., Kanaya H., Hayashi K., Konno T. (2012). Stress-induced takotsubo cardiomyopathy complicated with wall rupture and thrombus formation. Int. J. Cardiol..

[23] Kumar S., Kaushik S., Nautiyal A., Mostow N., Lazar J.M. (2012). Pathology findings mimicking acute myocardial infarction in a case of Takotsubo cardiomyopathy complicated by cardiac rupture. J. Cardiovasc. Med..

[24] Y-Hassan S. (2014). Cardiac rupture in a patient with Takotsubo syndrome triggered by acute myocardial infarction: Two messages. Int. J. Cardiol..

[25] Indorato F., Akashi Y.J., Rossitto C., Raffino C., Bartoloni G. (2015). Takotsubo cardiomyopathy associated with rupture of the left ventricular apex: Assessment of histopathological features of a fatal case and literature review. Forensic. Sci. Med. Pathol..

[26] Aikawa T., Sakakibara M., Takahashi M., Asakawa K., Dannoura Y., Makino T., Koya T., Tsutsui H. (2015). Critical takotsubo cardiomyopathy complicated by ventricular septal perforation. Intern. Med..

[27] Showkathali R., Dworakowski R., MacCarthy P. (2015). Catastrophic ruptured Takotsubo cardiomyopathy. J. Cardiovasc. Med..

[28] Miyake K., Funatsu T., Kondoh H., Taniguchi K. (2016). Rare Complication of Takotsubo Cardiomyopathy: Ventricular Septal Perforation with Septal Dissection. J. Card. Surg..

[29] Zalewska-Adamiec M., Bachórzewska-Gajewska H., Kożuch M., Frank M., Hirnle T., Dobrzycki S. (2016). Cardiac rupture in takotsubo cardiomyopathy treated surgically. Postepy. Kardiol. Interwencyjnej..

[30] Pepe M., Paradies V., Bortone A., De Cillis E., Cafaro A., Acquaviva T., Masi F., Quagliara D., Favale S. (2016). ‘Broken-heart’ syndrome: Ventricular septal perforation in a takotsubo cardiomyopathy. Future Cardiol..

[31] Sung J.M., Hong S.J., Chung I.H., Lee H.Y., Lee J.H., Kim H.J., Byun Y.S., Kim B.O., Rhee K.J. (2017). Rupture of Right Ventricular Free Wall Following Ventricular Septal Rupture in Takotsubo Cardiomyopathy with Right Ventricular Involvement. Yonsei Med. J..

[32] Mitchell A., Marquis F. (2017). Can takotsubo cardiomyopathy be diagnosed by autopsy? Report of a presumed case presenting as cardiac rupture. BMC Clin. Pathol..

[33] Kudaiberdiev T., Akhmedova I., Imanalieva G., Abdildaev I., Jooshev K., Ashimov J., Mirzabekov A., Gaybildaev J. (2017). Surgical treatment of left ventricular wall rupture, regarded as a consequence of Takotsubo cardiomyopathy. SAGE Open. Med. Case Rep..

[34] Iskander M., Abugroun A., Shehata K., Iskander F., Iskander A. (2018). Takotsubo Cardiomyopathy-Induced Cardiac Free Wall Rupture: A Case Report and Review of Literature. Cardiol. Res..

[35] Narita M., Sakakura K., Ohashi J., Ibe T., Yamamoto K., Wada H., Momomura S.I., Fujita H. (2019). Medically Treated Ventricular Septal Perforation Caused by Takotsubo Cardiomyopathy. Int. Heart. J..

[36] Tsuji M., Isogai T., Okabe Y., Nishimura Y., Itagaki S., Enatsu K., Hisagi M., Nonaka T., Ninomiya M., Otsuka T. (2018). Ventricular Septal Perforation: A Rare but Life-Threatening Complication Associated with Takotsubo Syndrome. Intern. Med..

[37] De Manna N.D., Bellamoli M., Santoro F., Vinco G., Pilati M., Ribichini F., Faggian G., Milano A.D. (2019). Midventricular Takotsubo cardiomyopathy complicated by a ventricular septal rupture: A surgical management. J. Cardiovasc. Med..

[38] Dalia T., Amr B.S., Agrawal A., Gautam A., Sethapati V.R., Kvapil J. (2019). A Rare Case of Sudden Death in a Patient with Takotsubo Cardiomyopathy Secondary to Cardiac Rupture. Case Rep. Cardiol..

[39] Zhukova N.S., Merkulova I.N., Shakhnovich R.M., Merkulov E.V., Osiev A.G., Pevzner D.V., Sukhinina T.S., Staroverov I.I. (2019). Endovascular closure of a ventricular septal defect from Takotsubo Syndrome. Ter. Arkh..

[40] Webster K.T., Apridonidze T., Mopala P.R., Sherman A.E., Potakamuri L.N., Storms D.R., Kono A.T., Mohanty B.D. (2019). Stress-Induced Cardiomyopathy Complicated by Dynamic Left Ventricular Outflow Obstruction, Cardiogenic Shock, and Ventricular Septal Rupture. Can. J. Cardiol..

[41] Al-Tkrit A., Mekaiel A., Aneeb M., Alawawdeh F., Mangla A. (2020). Left Ventricular Free Wall Rupture in Broken-Heart Syndrome: A Fatal Complication. Cureus.

[42] Mathew A., Berry E., Tirou M., Kumar P. (2020). Left ventricular rupture: A rare complication and an unusual presentation. BMJ Case. Rep..

[43] Kawai S., Suzuki H., Yamaguchi H., Tanaka K., Sawada H., Aizawa T., Watanabe M., Tamura T., Umawatari K., Kawata M. (2000). Ampulla cardiomyopathy (‘Takotsubo’ cardiomyopathy)—Reversible left ventricular dysfunction: With ST segment elevation. Jpn. Circ. J..

[44] Kurisu S., Sato H., Kawagoe T., Ishihara M., Shimatani Y., Nishioka K., Kono Y., Umemura T., Nakamura S. (2002). Tako-tsubo-like left ventricular dysfunction with ST-segment elevation: A novel cardiac syndrome mimicking acute myocardial infarction. Am. Heart J..

[45] Ptaszynska-Kopczynska K., Sobolewska D., Kozuch M., Dobrzycki S., Sobkowicz B., Hirnle T., Musial W., Kaminski K. (2011). Efficasy of invasive treatment and the occurrence of cardiac rupture in acute of ST-elevation myocardial infarction. Kardiol. Pol..

